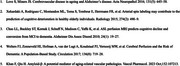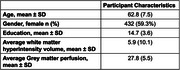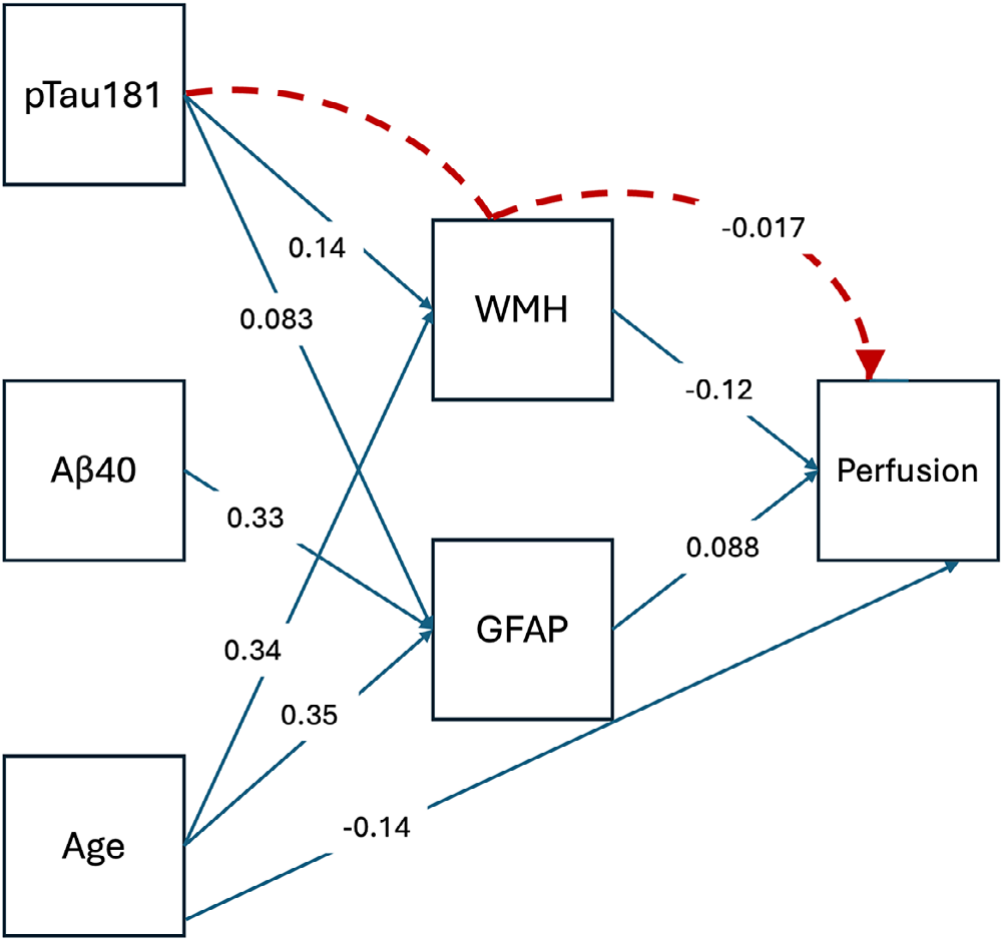# The effects of phosphorylated tau, amyloid‐β, neuroinflammation and cerebral white matter disease on cerebral perfusion: A path analysis study

**DOI:** 10.1002/alz70856_102462

**Published:** 2025-12-24

**Authors:** Ashwati Vipin, Gurveen Kaur Sandhu, Rasyiqah Binte Shaik Mohamed Salim, Nagaendran Kandiah

**Affiliations:** ^1^ Lee Kong Chian School of Medicine, Nanyang Technological University, Singapore, Singapore; ^2^ Dementia Research Centre (Singapore), Lee Kong Chian School of Medicine, Nanyang Technological University, Singapore, Singapore; ^3^ Dementia Research Centre (Singapore), Lee Kong Chian School of Medicine, Nanyang Technological University, Singapore 308232, Singapore, Singapore; ^4^ Neuroscience and Mental Health Programme, Lee Kong Chian School of Medicine, Nanyang Technological University, Singapore, Singapore

## Abstract

**Background:**

Reduced cerebral perfusion is a common pathological alteration in mild cognitive impairment and dementia[1]. Lower grey matter(GM) perfusion is predictive of cognitive decline[2] and conversion from mild cognitive impairment to dementia[3]. Thus, quantification of GM perfusion can be an important candidate biomarker of dementia risk[4]. Additionally, Aβ1‐40 as well as neuroinflammation have also been shown to play a significant role in vascular aging[5]. However, the link between CBF alterations and primary pathologies including Aβ1‐40 and fibrillar tau remains unclear. To address this gap, we sought to examine the impact of plasma Aβ, phosphorylated‐tau 181 (*p*‐tau181) and age on cerebral perfusion through pathways involving white matter hyperintensities burden (WMH) and the neuroinflammation marker, glial fibrillary acidic protein (GFAP).

**Method:**

Study participants belonged to the community‐based Biomarker and Cognition Cohort Study in Singapore and underwent cross‐sectional structural(T1)‐functional (Arterial spin labelling perfusion) MRI. Plasma‐based SIMOA assessment of *p*‐tau181, Aβ1‐40 and GFAP was carried out. Path analyses was constructed using RStudio lavaan package. Key predictors included *p*‐tau181, Aβ1‐40, age‐at‐visit with global grey matter cerebral perfusion as the main outcome variable. Log‐transformed WMH volume and plasma GFAP were included as mediators. Direct and indirect paths were defined. Missing values were imputed using mean imputation.

**Result:**

729 participants (mean age 62.8 years; 59.3% female sex) were included. Path analysis demonstrated a good model fit with a comparative fit index of 0.986 and Standardized Root Mean Square Residual of 0.014. Significant paths revealed *p*‐tau181 and age‐at‐visit being strong predictors of both GFAP and WMH, with logWMH negatively affecting GM perfusion. Aβ1‐40 was also a positive predictor of GFAP. Notably, the indirect effect of *p*‐tau181 on GM perfusion through logWMH was significant and negative. There were no indirect effects of *p*‐tau181 on GM perfusion through GFAP. There were also no indirect effects of Aβ1‐40 on GM perfusion through GFAP or WMH.

**Conclusion:**

This study attempts to examine simultaneous direct and indirect causal pathways between Aβ1‐40, *p*‐tau181 and age‐at‐visit on GM perfusion through GFAP and WMH. Our finding of indirect effect of *p*‐tau181 on GM hypoperfusion suggests a causal downstream effect of elevated *p*‐tau181 resulting in reduced GM perfusion through WMH.